# Hahnemann’s Defence of the Organon of Rational Medicine

**Published:** 1896-11

**Authors:** 


### BOOK NOTICES.

HAHNEMANN'S DEFENCE OF THE ORGANON OF RATIONAL MEDICINE AND OF HIS PREVIOUS HOMŒOPATHIC WORKS AGAINST THE ATTACKS OF PROF. HECKER. An Explanatory Commentary Upon the Homœopathic System. Translated by R. E. Dudgeon, M. D. Philadelphia: Bœricke & Tafel, 1896. Price, $1.10 net.

In this little volume Hahnemann takes up systematically the attacks made upon Homœopathy by Professor Hecker, of Dresden, "one of the most renowned authorities in medicine then existing, repeated from time to time during nearly fifteen years, and becoming ever more rancorous and calumnious as Hahnemann's system was slowly evolved and disclosed in his published works."

The work was "professedly written by Hahnemann's son Friederich," but it was undoubtedly the creation of the master himself. This is conclusively proved by Hahnemann's letter to the publisher, which Dr. Dudgeon has translated and incorporated in an "Introduction by the Translator," which explains the object of the book.

This book should be in the hands of every homœopathic physician in the land, not alone for the reason that it is a production of Hahnemann, but because, in defending his system, the master gives a great many new points of view, which enable the reader, especially if he be a student new to the subject, to get a clearer comprehension of the principle, and to remove many misapprehensions resulting from the talk of prejudiced persons.

Those who are older in the study of Homœopathy must also get valuable side-lights, so to speak, upon the principle, which must be a welcome addition to their stock of information and a desirable dissipator of many doubts as to the reasonableness of Hahnemann's claims.

The book was issued early in the present year, and should have been noticed in these pages long ago. The number of books that lie upon the editorial table, however, and the time required to read these books intelligently that a just criticism may be given, compel the delay.

